# High frequency of radiological differential responses with poly(ADP-Ribose) polymerase (PARP) inhibitor therapy

**DOI:** 10.18632/oncotarget.22303

**Published:** 2017-11-06

**Authors:** Raquel Perez-Lopez, Desam Roda, Begona Jimenez, Jessica Brown, Joaquin Mateo, Suzanne Carreira, Juanita Lopez, Udai Banerji, L. Rhoda Molife, Dow-Mu Koh, Stan B. Kaye, Johann S. de Bono, Nina Tunariu, Timothy A. Yap

**Affiliations:** ^1^ The Institute of Cancer Research, London, United Kingdom; ^2^ Royal Marsden NHS Foundation Trust, London, United Kingdom

**Keywords:** PARP inhibitors, *BRCA1* and *BRCA2* mutations, radiological differential responses

## Abstract

Despite impressive clinical activity in patients with germline *BRCA1* and *BRCA2 (BRCA1/2)* mutant cancers, antitumor responses to poly(ADP-Ribose) polymerase (PARP) inhibitors are variable. We set out to assess the rate of intrapatient radiological differential responses (RDR) to PARP inhibitors, its correlation with patient outcomes, and the identification of factors associated with RDR. We retrospectively reviewed all patients with advanced cancers from five early phase PARP inhibitor monotherapy trials. 113 patients (ovarian cancers 57.5%; breast cancers 23.9%) were included in this retrospective study; 46 (40.7%) patients developed RDR on PARP inhibitor monotherapy. We identified two patterns of RDR: early RDR (1st or 2nd on-treatment scans) in 69.6% of patients, and late RDR (penultimate or final scans) in 30.4% of patients. Early RDR was associated with shorter time to progression (TTP) (225 vs 367 days, HR:0.59, 95%CI 0.36-0.98; p=0.04) and overall survival (OS) (499 vs 857 days; HR:0.47, 95%CI 0.27-0.82, p=0.006). Seventy-nine (69.9%) patients had known germline *BRCA1/2* mutations; 49.4% of these *BRCA1/2* mutation carriers developed RDR versus 20.6% of patients with unknown or wildtype *BRCA1/2* status. Harboring germline *BRCA1/2* mutations was independently predictive for RDR (RR:2.93, 95% CI 1.08-7.90, p=0.03). Patients with germline *BRCA1* mutations had worse TTP and OS than *BRCA2* mutation carriers (212 vs 406 days, HR:0.58, 95% CI 0.36-0.94, p=0.023 and 515 vs 937 days; HR:0.49, 95% CI 0.29-0.83; p=0.007). RDR with PARP inhibitors are frequent, particularly in germline *BRCA1/2* mutation carriers. These findings have clinical implications for patient outcomes and may reflect underlying intrapatient genomic heterogeneity.

## INTRODUCTION

Poly(ADP-Ribose) polymerase (PARP) inhibitors result in impressive clinical activity in patients with germline *BRCA1* and *BRCA2 (BRCA1/2)* mutant cancers through a single agent therapeutic strategy based on the concept of tumor-specific synthetic lethality [1-5]. The PARP inhibitor olaparib (AstraZeneca) was recently approved by the Food and Drug Administration (FDA) for the treatment of germline *BRCA1/2* mutated patients with advanced ovarian cancers who have been treated with three or more prior lines of chemotherapy, and in the maintenance setting. Improvements in patient outcomes have also been recently demonstrated with another PARP inhibitor niraparib as maintenance therapy post-platinum chemotherapy [6], and in the advanced setting with rucaparib [7], also leading to FDA approval for both drugs. Despite these therapeutic advances in patients with germline *BRCA1/2* mutant cancers, PARP inhibitor therapy still results in variable responses between patients, and secondary resistance to therapy is inevitable.

The Response Evaluation Criteria in Solid Tumors (RECIST) introduced in 2000 and modified in 2009 are the standard FDA-approved validated criteria used to objectively assess antitumor responses to anticancer therapies based on radiological assessments [8, 9]. RECIST was designed and successfully used to assess antitumor responses to cytotoxic agents; however, the introduction of novel therapies has highlighted limitations with RECIST, particularly when they are used to assess responses to molecularly targeted agents, where treatments are matched to specific molecular features [10-12]. In addition, radiological differential responses (RDR) or mixed responses, with the co-existence of disease progression in one or more lesions and complete response (CR) or partial response (PR) in other lesion(s) in the same patient at the same timepoint have also been described in patients receiving molecularly targeted agents [13-16]. For example, differential responses observed in patients with melanoma treated with BRAF inhibitors have been reported to occur in the order of 29-38% of patients [13, 14]. Such discordant intrapatient variations in antitumor responses to targeted therapies may, in part, be due to underlying intrapatient molecular heterogeneity and tumor clonal evolution, which have both been previously described in advanced cancers [13, 17, 18].

While treating patients with advanced solid tumors with single agent PARP inhibitors within the context of early phase clinical trials, we observed an anecdotally high frequency of patients with RDR on treatment, including those with germline *BRCA1/2* mutation cancers. We therefore designed and conducted a retrospective study of patients with advanced cancers treated with PARP inhibitors within clinical trials. The primary endpoint of this study was to assess the overall rate of inter and intra organ RDR and its correlation with treatment outcome. We specifically sought to assess if there was a higher frequency of RDR with PARP inhibitor treatment in patients with germline *BRCA1/2* mutation cancers versus those with wildtype or unknown *BRCA1/2* mutation status. In addition, we explored other baseline patient characteristics that may be predictive for the development of RDR with PARP inhibitors in our series of patients. Finally, we assessed if RECIST 1.1 was able to capture intrapatient heterogeneous responses to PARP inhibitors.

## RESULTS

### Baseline characteristics

A total of 113 patients with advanced solid tumors were treated with PARP inhibitors as monotherapy within the context of five clinical trials between March 2006 and March 2014, and were eligible for this study. The median age at trial entry was 53.7 years (range: 18-82 years); 99 of 113 (87.6%) patients were women. The most frequent sites of metastatic disease at baseline included: lymph nodes in 66 of 113 (58.4%) patients, peritoneum in 63 of 113 (55.8%) patients, liver in 23 of 113 (20.4%) patients and lung in 19 of 113 (16.8%) patients. None of the patients had previously been exposed to PARP inhibitors prior to participating in these trials. 79 of 113 (69.9%) patients had known germline *BRCA1/2* mutations, while 34 of 113 (30.1%) were *BRCA1/2* wildtype or unknown (Table 1). Other baseline characteristics are summarized in Table 1. The reasons for trial discontinuation were disease progression in 106 patients (93.8%), and toxicity in 7 patients (6.2%).

**Table 1:** Baseline characteristics of the study population (n=113)

Baseline characteristic	
***BRCA1/2* status**	**N (%)**
Germline *BRCA1/2* mutation	79 (69.9%)
*BRCA1/2* wildtype or unknown	34 (30.1%)
**Gender**	**N (%)**
Female	99 (87.6%)
Male	14 (12.4%)
**Age**	**median (range)**
	53.7 (19-82)
**ECOG Performance Status**	**N (%)**
0	31 (27.4%)
1	81 (71.7%)
2	1 (0.9%)
**Number of previous lines of chemotherapy**	**median (range)**
	3 (1-8)
**Primary Tumor type**	**N (%)**
Breast	27 (23.9%)
Triple negative	11
ER positive	11
HER2 positive	7
Ovarian	65 (57.5%)
High grade serous	48
Serous papillary	13
Other	4
Primary peritoneal	4 (3.5%)
Endometrial	8 (7.1%)
Colorectal	2 (1.8%)
Sarcoma	2 (1.8%)
Prostate	2 (1.8%)
Lung	3 (2.7%)

Abbreviations: ER: Estrogen receptor; HER2: human epidermal growth factor receptor 2.

### RDR in overall population

#### Incidence and timing of radiological differential responses (RDR)

Overall, 46 of 113 (40.7%) patients developed RDR during the course of treatment with a PARP inhibitor. Of the two most frequent tumor types included in this study, 35 of 65 (53.8%) patients with advanced ovarian cancers had RDR, while 5 of 27 (18.5%) patients with advanced breast cancers developed RDR. Overall, we observed RDR at two specific timepoints during treatment: early RDR (1st or 2nd on-treatment scans) in 32 of 46 (69.6%) patients and late RDR (penultimate or final scans) in 14 of 46 (30.4%) (Supplementary Table 2).

**Figure 1:**
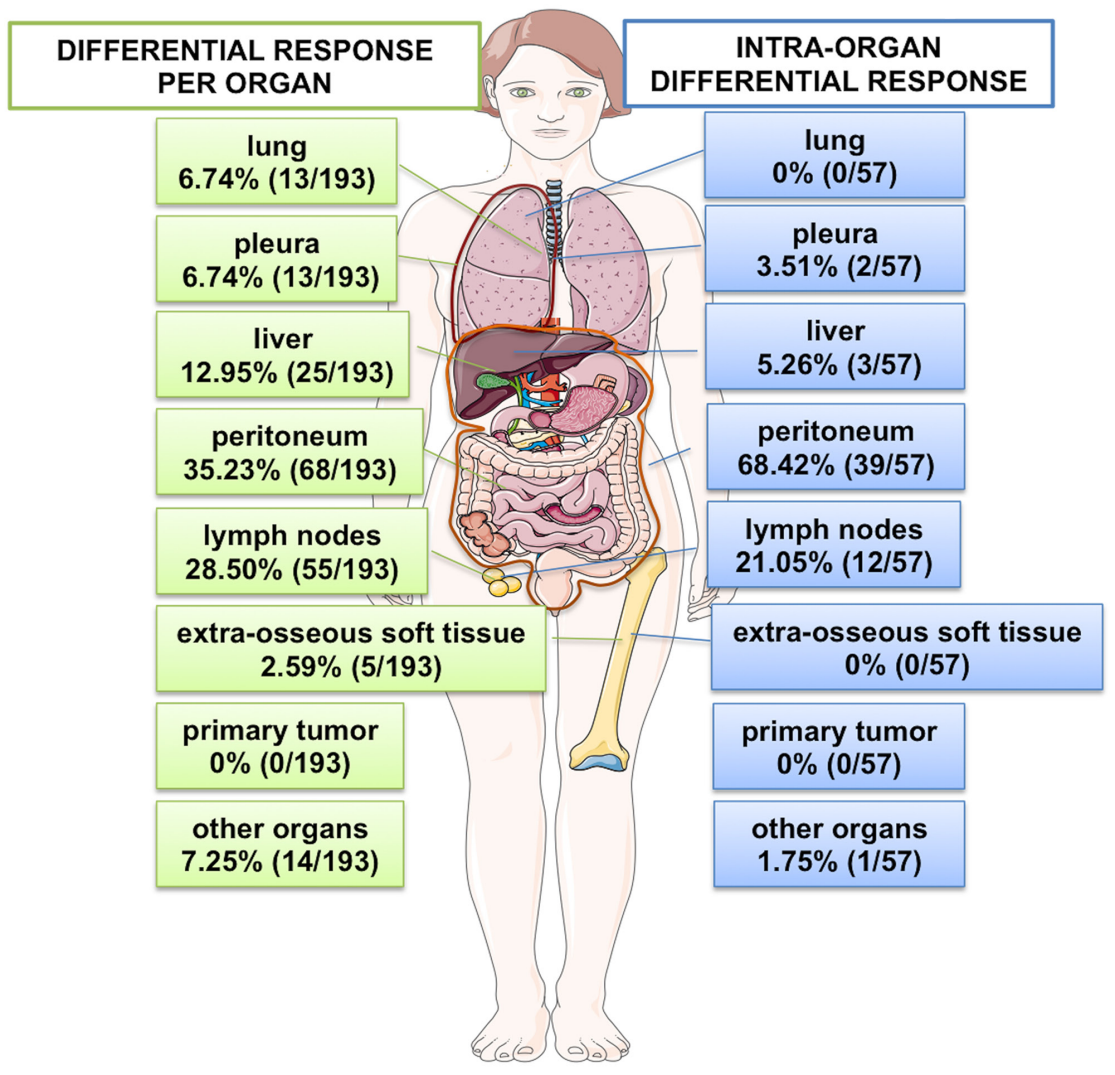
Inter and intra organ RDR Anatomical distribution of lesions demonstrating inter organ RDR are shown on the left panel (total number of lesions demonstrating RDR: n=193) and those demonstrating intra organ RDR are shown on the right panel (total number of patients with intra organ RDR: n=57).

A total of 830 lesions were reviewed during this study, of which 193 (23.3%) were shown to demonstrate RDR on restaging assessments during PARP inhibitor treatment when compared to baseline imaging; new lesions occurring while on treatment compared to baseline were also included in this study. The most frequent locations where RDR occurred included the peritoneum (68 of 193 [35.2%]), lymph nodes (55 of 193 [28.5%]), liver (25 of 193 [13.0%]), lung (13 of 193 [6.7%]) or pleura (13 of 193 [6.7%]) (Figure 1).

We detected 57 events involving intra-organ RDR; some patients presented with RDR in more than one organ at the same time. Overall, the incidence of intra-organ RDR was higher in the peritoneum (39 of 57 [68.4%]), followed by lymph nodes (12 of 57 [21.1%]) and liver (3 of 57 [5.3%]) (Figure 1 and Supplementary Figure 1).

#### Radiological differential responses and patient outcomes

**Figure 2:**
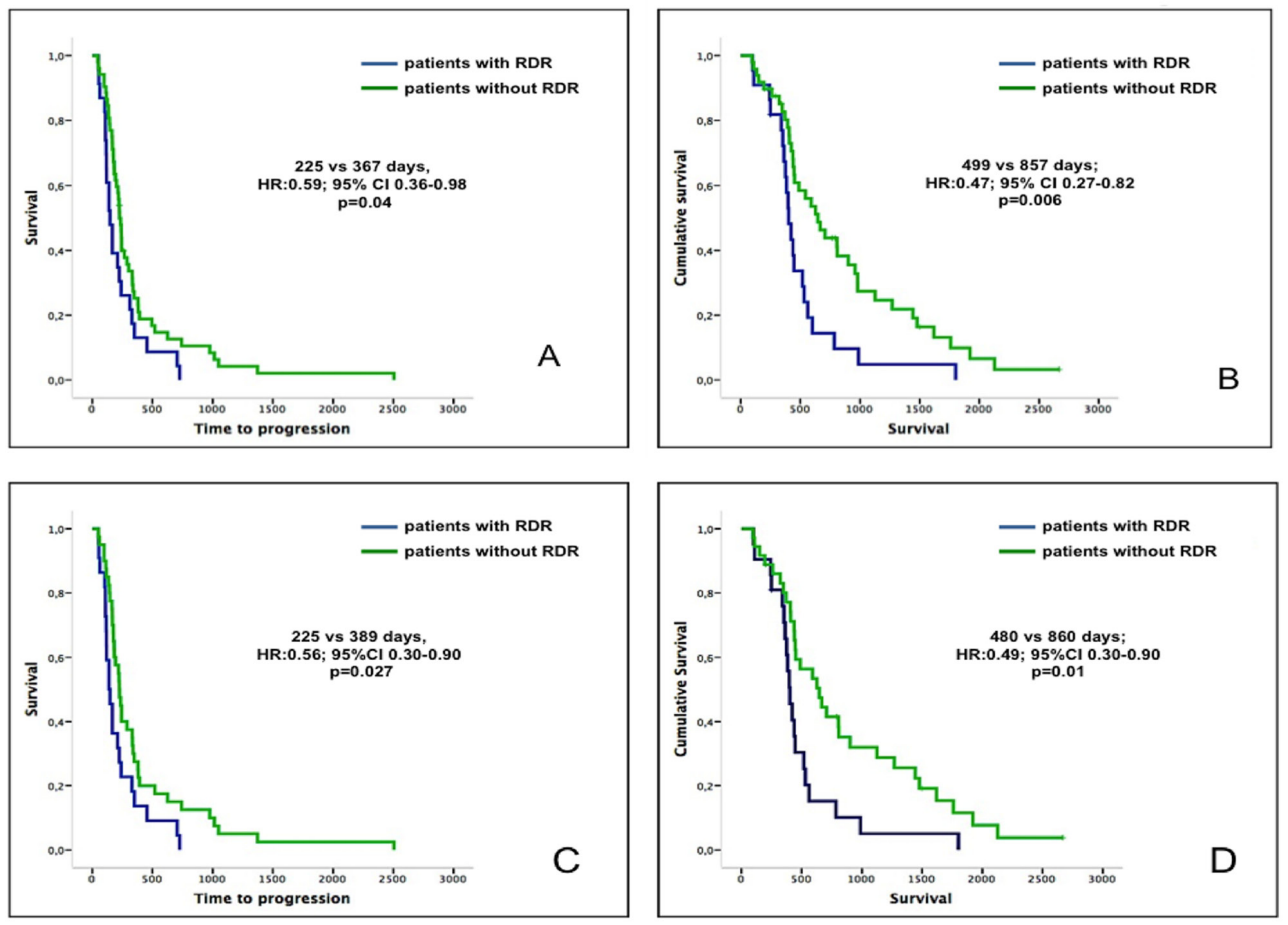
TTP and OS of patients with early RDR vs no early RDR **(A** and **B)**: TTP and survival of patients with early RDR versus those patients without RDR in early scans (1^st^ and 2^nd^ on-treatment scans). **(C** and **D)**: TTP and survival of patients harboring germline *BRCA1/2* mutant tumors with early RDR versus those without RDR in early scans (1^st^ and 2^nd^ on-treatment scans). Patients with early RDR are represented by the blue curves, while patients without early RDR are represented by the green curves.

Patients who developed early RDR and continued on treatment (based on overall PR or stable disease (SD) according to RECIST1.1) had a significantly shorter median TTP, in contrast to patients without RDR (225 vs 367 days, HR: 0.59 95% CI 0.36-0.98; p=0.04). Moreover, the median OS in patients who developed an early RDR was significantly worse when compared to the non-RDR subgroup (499 vs 857 days; HR: 0.47 95% CI 0.27-0.82, p=0.006) (Figure 2A and 2B). An example of a patient with early RDR and PR by RECIST criteria 1.1 is shown in Supplementary Figure 2. Median TTP and OS were similar between patients who developed a late RDR versus those who did not have a RDR (589 vs 424 days, HR 0.85, 95% CI 0.78-1.30, p=0.48).

### RDR and classification by RECIST 1.1

**Figure 3:**
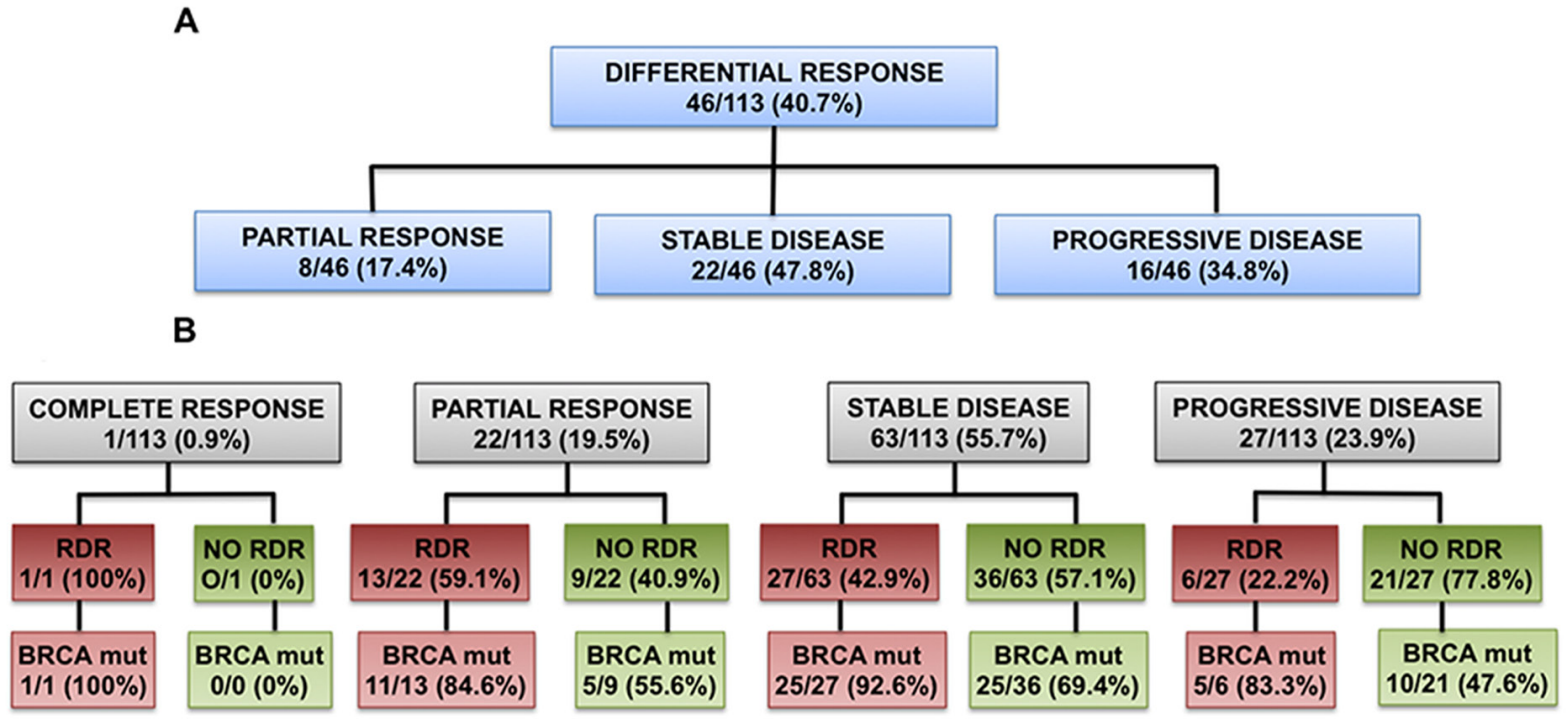
RDR and classification by RECIST 1.1 **(A)**: RECIST 1.1 assessment at the time of RDR occurrence in the 46 patients who developed RDR. **(B)**: Incidence of RDR when considering their best response according to RECIST 1.1 (RDR and best response by RECIST 1.1 may have happened at different time-points).

In the 46 patients who developed RDR, their corresponding RECIST 1.1 assessment at the time of RDR occurrence was SD in 22 of 46 (47.8%) patients, PD in 16 of 46 (34.8%) patients and PR in 8 of 46 (17.4%) patients (Figure 3A).

In our series of patients, when considering their best response according to RECIST 1.1, 22 of 113 (19.5%) patients had a PR, of which 13 of 22 (59.1%) patients were deemed to have RDR at some point during PARP inhibitor treatment (i.e. before or after their best response of PR); 1 of 113 (0.9%) patients achieved a CR, but subsequently developed a late RDR during PARP inhibitor treatment as per our study criteria; 63 of 113 (55.8%) patients had a best response of SD, of which 27 of 63 (42.9%) had RDR at some point during their treatment (i.e. before or after their best response of SD); 27 of 113 patients (23.9%) had PD, of which 6 of 27 (22.2%) had RDR prior to developing PD (Figure 3B).

Importantly, the TTP and OS of patients who developed a CR or PR by RECIST 1.1 were not significantly different between patients who did or did not develop RDR (332 vs 329 days, HR: 0.79, 95% CI 0.32-1.95, p=0.60 and 980 vs 805 days, HR: 1.4, 95% CI 0.44-4.71, p=0.55). The TTP and OS of patients who achieved RECIST SD were also not significantly different between both groups of patients (147 vs 148 days, HR: 1.01, 95% CI 0.61-1.67, p=0.98 and 447 vs 398 days, HR: 1.5, 95% CI 0.83-2.56, p=0.19).

### Predictive factors for the development of RDR

We assessed multiple baseline characteristics to establish if any clinical, molecular or laboratory variables were predictive for the development of RDR during treatment. The presence of peritoneal disease, harboring a primary ovarian cancer, and germline *BRCA1/2* mutations were found to significantly predict for RDR in a univariate logistic regression analysis (Table 2). Baseline ECOG performance status, the Royal Marsden Hospital prognostic score [19], greater than three prior lines of antitumor therapies, and platinum sensitivity did not significantly predict the development of RDR in the univariate analysis.

**Table 2:** Univariate and multivariate analysis of predictive factors for RDR

Univariate analysis	RR (95% CI)	P value
Baseline ECOG performance status	1.10 (0.25-3.4)	p=0.19
> 3 prior lines of antitumor therapy	0.87 (0.54-1.46)	p=0.57
RMH prognostic score	0.77 (0.40-1.39)	p=0.48
Platinum sensitive disease	0.55 (0.34-1.46)	p=0.19
Disease location:		
Visceral disease	0.79 (0.5-1.63)	p=0.37
Liver disease	1.85 (0.74-4.67)	p=0.24
Nodal disease	0.80 (0.50-1.27)	p=0.44
Peritoneal disease	1.36 (1.01-1.83)	p=0.05
Tumor type (ovarian cancer vs other tumors)	1.90 (1.10-3.4)	p=0.01
*BRCA1/2* mutation	2.70 (1.40-6.50)	p=0.004
*BRCA1* vs *BRCA2* mutations	2.05 (1.17-4.48)	p= 0.015

Multivariate analysis	RR (95% CI)	P value
*BRCA1/2* mutation	2.93 (1.08-7.91)	p=0.03
Tumor type (ovarian cancer vs other tumors)	2.00 (0.84-4.77)	p=0.12

A multivariate analysis subsequently undertaken then confirmed germline *BRCA1/2* mutations as the only independent predictive factor for the development of RDR (RR 2.93, 95% CI 1.08-7.91, p=0.03). Among patients with germline *BRCA1/2* mutations, *BRCA1* mutations contributed most significantly to this risk of developing RDR (RR: 2.05 [1.17-4.48]; p=0.015) (Table 2).

### RDR among patients harboring germline *BRCA1/2* mutant tumors

39 of 79 (49.4%) germline *BRCA1/2* mutant patients with advanced cancers developed RDR while receiving PARP inhibitor monotherapy, versus 7 of 34 (20.6%) patients with unknown or wildtype *BRCA1/2* status (RR: 2.70,95% CI 1.40-6.50, p=0.004). Patients with germline *BRCA1/2* mutant cancers who developed an early RDR and continued on therapy (because of RECIST PR or SD) had significantly shorter TTP and OS when compared to those who did not have RDR (225 vs 389 days, HR: 0.56, 95% CI 0.30-0.90, p=0.027, and 480 vs 860 days, HR: 0.49, 95% CI 0.30-0.90, p=0.01, respectively) (Figure 2C and 2D). In addition, patients with germline *BRCA1/2* mutant tumors who had a late RDR did not have a significantly different TTP or OS in contrast to patients who did not develop RDR (449 vs 665 days, HR: 0.89, 95%CI 0.80-1.20, p=0.42 and 1004 vs 1059 days, HR: 0.77, 95%CI 0.29-2.00, p=0.59).

### Germline *BRCA1* vs *BRCA2* mutations and patient outcomes

When comparing baseline characteristics between our series of patients who harbored germline *BRCA1* mutant tumors versus those with *BRCA2* mutant cancers, both groups were well balanced for baseline characteristics, including tumor type, platinum sensitivity, baseline sites of metastatic disease, and number of prior lines of chemotherapy (Supplementary Tables 3 and 4).

**Figure 4:**
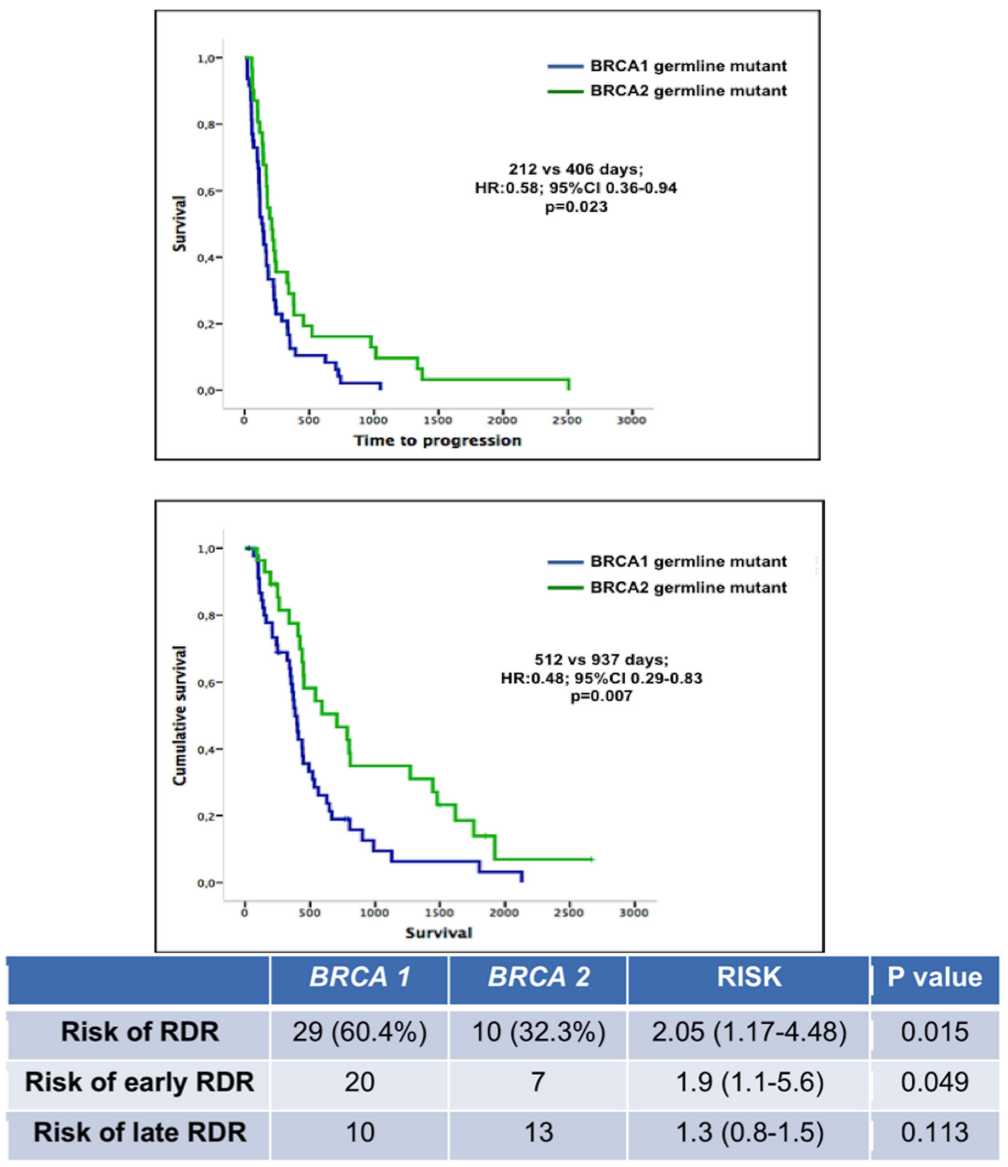
TTP and OS of patients harboring germline *BRCA1* and *BRCA2* mutations Patients with germline *BRCA1* mutant cancers had a significantly higher risk of RDR and early RDR, but a similar risk of late RDR compared to patients with germline *BRCA2* mutant cancers.

We compared the outcomes of patients with germline *BRCA1* mutations with those harboring germline *BRCA2* mutations when treated with PARP inhibitor monotherapy. TTP for *BRCA1* mutant patients was significantly shorter compared to *BRCA2* mutation carriers (212 vs 406 days, HR: 0.58, 95% CI 0.36-0.94, p=0.023). There was also a significantly reduced OS for patients with germline *BRCA1* mutant cancers versus those harboring *BRCA2* mutant cancers (515 vs 937 days; HR: 0.49, 95% CI 0.29-0.83; p=0.007) (Figure 4).

Patients with germline *BRCA1* mutation demonstrated an increased risk of RDR compared to those harboring *BRCA2* mutations (60% vs 32%, RR 2.1, 95% CI 1.20-4.5, p=0.015). This risk was increased for early RDR (RR 1.9, 95% CI 1.20-5.6, p=0.04), rather than late RDR (RR 1.3, 95% CI 0.8-1.5, p=0.11). In addition, among patients with germline *BRCA1* mutations, those who developed early RDR demonstrated a shorter TTP in contrast to those without RDR (184 vs 334 days, p=0.025). There was also a trend towards reduced TTP for patients with germline *BRCA2* mutant cancers who developed RDR compared to germline *BRCA2* mutant patients who did not have RDR (320 vs 494 days; p=0.045).

### RDR and intrapatient biological heterogeneity

**Figure 5:**
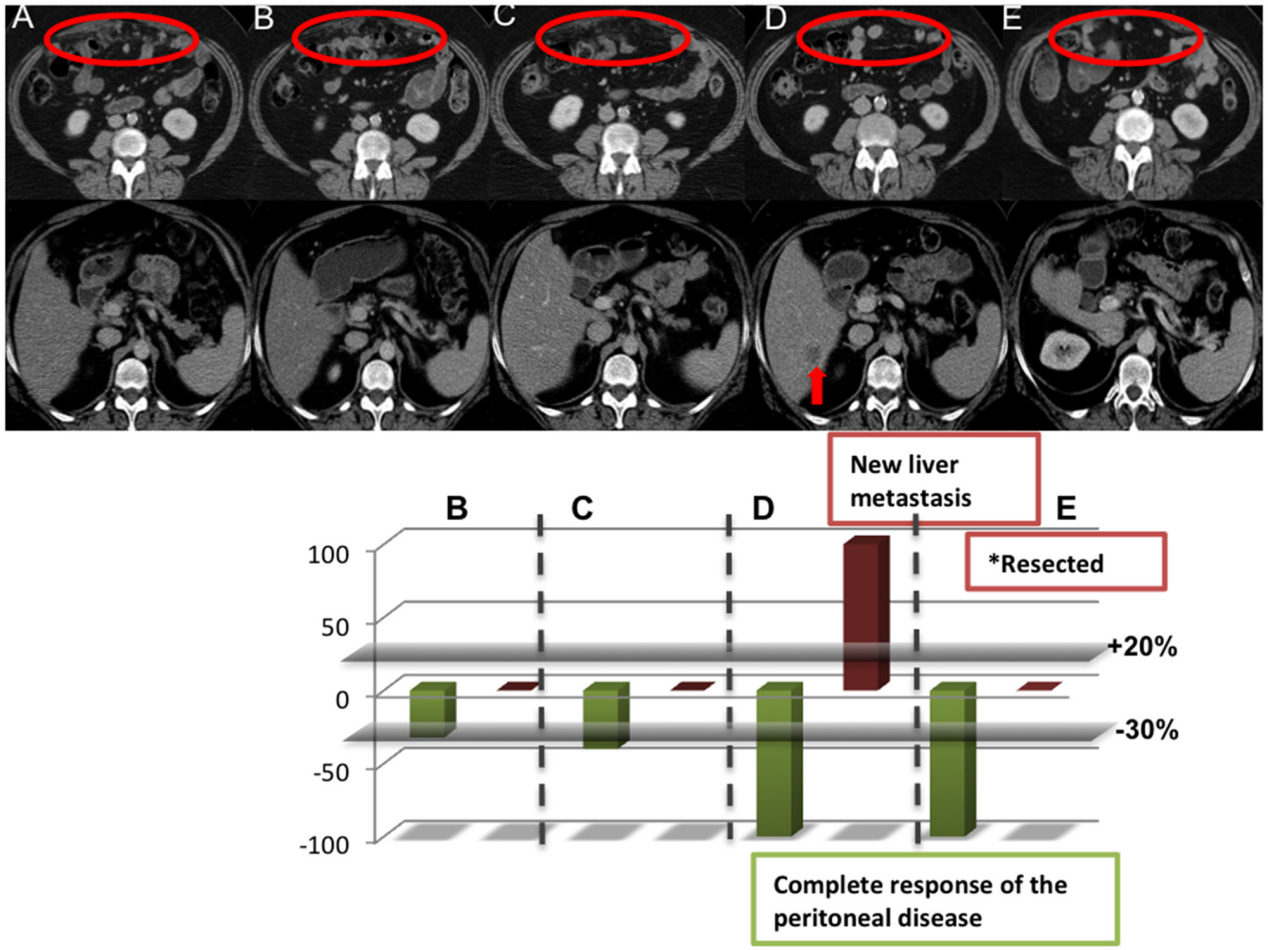
Tumor characterization of a PARP inhibitor responder with RDR Axial enhanced CT images in a 52-year-old female with germline BRCA2 mutation papillary serous ovarian carcinoma who commenced PARP inhibitor monotherapy within a phase I clinical trial in July 2007. **(A)** The baseline CT shows multiple peritoneal deposits measuring up to 25mm (circle) and no metastatic liver disease. **(B)** The 12 weeks and **(C)** 24 weeks CT show marked reduction in size of the peritoneal deposits (circle) and no liver metastases. **(D)** A subsequent CT in November 2013 showed an excellent response in the peritoneal disease with no CT evidence of peritoneal disease relapse or ascites but, with a new solitary liver metastasis in segment VI (arrow), which was subsequently surgically resected. **(E)** A subsequent CT in June 2015 shows maintained complete radiological response.

To assess the biological mechanisms underlying these intrapatient heterogeneous radiological outcomes, we molecularly characterized tumor samples collected from a patient who had developed RDR on PARP inhibitor therapy. She was a 52-year-old female with metastatic papillary serous ovarian cancer, who was known to harbor a deleterious germline *BRCA2* mutation (p.W563^*^). She initially started on PARP inhibitor monotherapy having progressed on standard lines of treatment for metastatic disease. Her baseline CT imaging showed multiple peritoneal metastases measuring up to 25mm and no hepatic metastases. After 3 months of PARP inhibitor monotherapy, she achieved a RECIST 1.1 CR with no evidence of peritoneal nodules or ascites. After 81 months of a sustained CR to PARP inhibitor monotherapy, CT imaging revealed a new solitary liver metastasis, despite a maintained response in her peritoneal disease (Figure 5). The patient subsequently underwent a liver metastasectomy, with histology of her resected lesion confirming metastatic papillary serous carcinoma of the ovary.

Targeted NGS was undertaken on her primary tumor sample and the liver metastasis surgical specimen. Germline DNA was extracted from saliva for targeted NGS. In the initial ovarian tumor, biallelic *BRCA2* inactivation was present, with the known germline p.W563^*^ *BRCA2* mutation and a second somatic frameshift p.K650fs^*^8 deletion detected at an allele frequency of 16.4% (105/639 reads).

In contrast, NGS of the liver metastasis revealed a deletion in chromosome 13q, including *BRCA2, RB1 and ERRC5*, accounting for the allele containing the germline *BRCA2* p.W563^*^ mutation, which was only present in 4% of sequencing reads (in a sample with 90% tumor content). The somatic frameshift p.K650fs^*^8 deletion was not detected in the liver metastasis (sequencing depth of the region: 116x). As a result, the liver metastasis contained one *BRCA2* allele that was wildtype, rendering the metastasis *BRCA2*-proficient, which was in keeping with the tumor growth observed despite ongoing PARP inhibitor treatment. We hypothesize that the liver metastasis arose from a subclone of the disease not containing the frameshift somatic mutation, which was heterogeneous within the primary tumor, and acquired a new deletion of 13q, eliminating the germline mutant allele.

After the liver metastasectomy surgery, the patient continued PARP inhibitor monotherapy for another 15 months, when she relapsed again in the liver and developed an enlarged retrocaval lymph node. Both lesions were treated effectively with chemoembolization and focal radiotherapy, while her peritoneal lesions, which would arguably continue to have *BRCA2* biallelic inactivation, continued to remain in response after 9 years of therapy.

## DISCUSSION

To our knowledge, this is the first study that evaluates the differential patterns of antitumor responses to PARP inhibitor monotherapy in patients with advanced solid tumors, including those with and without germline *BRCA1/2* mutations. Data from our study suggest that RDR is a prevalent phenomenon, occurring in 41% of patients treated with PARP inhibitor monotherapy in our series of patients with advanced solid tumors. We identified two patterns of RDR, and showed that patients who developed early RDR had worse TTP and OS, whereas outcomes were similar between patients who developed a late RDR compared with those who did not have a RDR. In addition, nearly 50% of *BRCA1/2* mutation carriers in our series of patients developed RDR while receiving PARP inhibitor monotherapy, compared to approximately 20% of patients with unknown or wildtype *BRCA1/*2 mutation status. This is a higher prevalence of RDR than that reported with other targeted agents [13, 14]. We also show through a multivariate analysis that germline *BRCA1/2* mutations were independently predictive for RDR. Furthermore, patients with *BRCA1* mutation cancers had worse TTP and OS compared to those with *BRCA2* mutation cancers, and our data suggest that this could potentially be related to an increased incidence of RDR. Finally, our long-term PARP inhibitor responder case study also illustrates that patients who develop RDR with an isolated area of clonal progression can receive local therapy and still continue to gain durable benefit from PARP inhibitor treatment.

RECIST 1.1 remains the standard criteria for antitumor response assessment in clinical trials. These radiological criteria were originally designed to assess the effects of cytotoxic chemotherapies rather than molecularly targeted agents, and are reliant on tumor shrinkage to demonstrate antitumor activity [8, 9]. In our study, of the 46 patients who developed RDR, their corresponding RECIST 1.1 assessment at the time of RDR occurrence was SD in 22 of 46 (47.8%) patients, progressive disease (PD) in 16 of 46 (34.8%) patients and PR in 8 of 46 (17.4%) patients. Our data demonstrate that RECIST criteria failed to accurately capture intra and inter organ radiological heterogeneity in response to PARP inhibitor monotherapy.

In our series of patients receiving PARP inhibitor monotherapy, developing early RDR was prognostic of a worse clinical outcome. The detection of early RDR may support undertaking short-term follow-up scans to confirm oligo-progressive disease, consideration of local therapy if feasible, conducting biopsies of progressing lesions for molecular profiling to potentially identify resistant subclones to guide a switch in antitumor therapy or to facilitate the design of rational combination strategies. A relatively less invasive alternative may be to monitor tumor clone dynamics by molecularly characterizing circulating tumor DNA from serially obtained plasma specimens using NGS. Interestingly, patients who developed a late RDR had similar patient outcomes as those who did not have a RDR. As illustrated by our long term PARP inhibitor responder case study and consistent with findings from other groups [20], patients who develop late RDR through oligometastatic disease recurrence should be considered for local antitumor treatment strategies if appropriate, such as surgery or cyberknife radiosurgery, to potentially enable patients to continue systemic disease control with PARP inhibitor therapy. Preclinical and early phase clinical studies have also now provided evidence for rational synergistic drug combinations involving PARP inhibitors, which could potentially aid in the control of secondary resistance [21].

NGS studies have shown that human malignancies exhibit intrapatient and even intratumor molecular heterogeneity, comprising distinct cellular populations with specific genetic features across different geographical regions within the same tumor [22]. Furthermore, tumor subclones with distinct mutational profiles have been shown to evolve temporally under specific microenvironmental pressures following Darwinian evolution patterns in response to anticancer treatments [17]. We present a case study of a long term PARP inhibitor responder - a germline *BRCA2* mutation carrier with advanced ovarian cancer – who despite achieving an excellent response to PARP inhibitor monotherapy, twice developed radiological disease progression of liver metastases, arguably secondary to the selection of a *BRCA2* proficient clone, potentially from an heterogeneous primary tumor [23, 24]. Durable responses to PARP inhibitors have been associated with the inactivation of both *BRCA1/2* alleles in the tumor; in this case, emergence of one putatively functional allele was detected at progression [25]. The patient benefited from salvage local therapy to the progressing lesions, and the patient continues to benefit from PARP inhibitor treatment for more than 10 years. While we acknowledge that this case may not explain all instances of RDR, it exemplifies how RDR can potentially indicate underlying intrapatient genomic heterogeneity and how exceptional responders can aid in our understanding of the molecular underpinnings of cancer. A recent study undertaken in patients with castration-resistant prostate cancers harboring *BRCA2* mutations demonstrated the polyclonal nature of resistant subclones [26]. Considering that patients in our cohort previously received platinum-based chemotherapies, which have been shown to favor the emergence of similar mechanisms of resistance as PARP inhibitors, this polyclonality may be relevant for the heterogeneous pattern of disease response and progression observed in our study [27].

When examining baseline clinical factors that were predictive for RDR, germline *BRCA1/2* mutations were found to be an independent risk factor for the development of RDR (p=0.03), with *BRCA1* mutations contributing most significantly to this increased risk (p=0.015). In addition, 39 of 79 (49.4%) patients with *BRCA1/2* mutant cancers developed RDR when receiving PARP inhibitor monotherapy, in contrast to 7 of 34 (20.6%) patients with unknown or wildtype *BRCA1/2* status (p=0.004). Consistent with previous studies [28-30], patients harboring germline *BRCA1* mutant tumors had a worse TTP (p=0.023) and shorter OS (p=0.007) to PARP inhibitor monotherapy compared to patients with germline *BRCA2* mutant cancers in our series of patients. Potential reasons for this difference could be the higher prevalence of patients with triple negative breast and high-grade serous ovarian cancers, which harbored germline *BRCA1* mutant tumors. Interestingly, a higher frequency of RDR and therefore potentially increased rates of subclonality, was observed in patients with germline *BRCA1* versus *BRCA2* mutant tumors, possibly contributing to the observed differences in sensitivity to PARP inhibitors. Lheureux and colleagues recently reported a higher prevalence of *BRCA1* mutant patients who had short term responses (less than 3 months) to olaparib compared to those with *BRCA2* mutations [31]. As observed in our series of patients, the higher incidence of early RDR in patients with *BRCA1* mutations was associated with shorter responses to PARP inhibitors. Nevertheless, both cohorts of patients with germline *BRCA1* and *BRCA2* mutant tumors in our study involved relatively small numbers of patients and these data will therefore need to be confirmed in larger cohorts of patients. We acknowledge that the retrospective nature of our study and the fact that the majority of patients included in our patient population had advanced ovarian cancers (57.5%) are limitations for the generalization of these data. However, the findings presented in this study are novel and support future prospective research involving larger numbers of patients, including the collection of multiple tumor samples from patients with different cancers to investigate the concordance between RDR and molecular intratumor heterogeneity.

In summary, our data demonstrate that RDR is a relatively frequent phenomenon among patients treated with PARP inhibitors, especially those with germline *BRCA1/2* mutant cancers. Also, germline *BRCA1/2* mutations were an independent predictive factor for the development of RDR, while patients harboring germline *BRCA1* mutant tumors had worse outcomes to PARP inhibitors relative to patients with *BRCA2* mutant cancers. We show that patients with early RDR had worse TTP and OS, whereas outcomes were similar between patients who developed late RDR compared with those who did not have RDR. These data suggest that RDR may potentially be used as a clinical indicator to predict therapeutic responses and be utilized to guide further investigations and/or appropriate management. Overall, these findings may reflect underlying intrapatient tumor heterogeneity, and have important clinical implications for patients receiving PARP inhibitors.

## MATERIALS AND METHODS

### Patients and treatment

This is a retrospective analysis of patients treated in five separate single agent PARP inhibitor phase I clinical trials in the Drug Development Unit at the Royal Marsden NHS Foundation Trust, London, England, UK. This study was approved by the Royal Marsden Hospital Committee for Clinical Research and Development. Only patients who received PARP inhibitors at biologically active monotherapy doses and who were assessed for response to therapy in these phase I clinical trials were entered into this study. Only patients with measurable disease by RECIST 1.1 on computed tomography (CT) or magnetic resonance imaging (MRI) at baseline were included. Patients with active secondary malignancies, those who failed entry screening for the trial, and/or those who did not receive any trial drug were excluded from this study. Data on patient and disease characteristic at trial entry were collected from electronic patient records. Platinum-sensitive cancer was defined as the recurrence of disease on platinum chemotherapy after more than 6 months, while platinum-resistant cancer was defined as the recurrence of disease on platinum chemotherapy after less than 6 months.

### Imaging response assessment methodology

All enrolled patients had CT (97 of 113 patients; 85.8%) or MRI (16 of 113 patients; 14.2%) scanning assessed at baseline and every 8-12 weeks throughout PARP inhibitor treatment according to specific trial protocols. Identical standard CT and MRI acquisition protocols were employed for all patients (contrast enhanced, portal venous phase, multidetector CT or 1.5T Siemens Avanto MRI, continuous acquisition, 5mm slice thickness). A maximum of five scans (CT, MRI) per patient were reviewed including: baseline scans, the first two on-treatment imaging studies and the final two scans undertaken before disease progression or discontinuation of treatment for any cause. For each patient, the same imaging modality (CT or MRI) was followed throughout the trial. Images were anonymised and made available for radiology review using the standard hospital SECTRA PACS (Picture Archiving and Communication System) IDS7™ workstations. The overall response assessment per RECIST 1.1 (CR, PR, SD or PD) and the respective clinical trial timepoint were recorded for each patient.

### Definitions and assessment of radiological differential responses (RDR)

Two board-certified radiologists with experience in oncological trial imaging (RPL: 3 years and NT: 8 years) were blinded to clinical and previous radiological assessment data, and both radiologists reviewed all cases independently. The radiological response to treatment was recorded for each lesion on all selected MRI/CT scans according to primary and metastatic sites of disease: lung, pleura, liver, peritoneum, soft tissue associated with bone metastases, other viscera and lymph nodes, which were assessed per location as supraclavicular, thoracic, abdominal, retroperitoneal or pelvic lymph nodes.

The radiological response for each lesion was classified according to the principles of RECIST 1.1: CR (disappearance of the lesion, or reduction in short axis to <10 mm in the case of a pathological lymph node), PR (a decrease by at least 30% of the long axis for visceral or soft tissue disease, and short axis for pathological lymph nodes), PD (an increase of at least 20% of the long axis in the case of visceral or soft tissue disease; and short axis in the case of pathological lymph nodes), or SD (when the lesion does not fulfill criteria for PR or PD).

RDR was defined as the co-existence of progression in one or more lesions and CR or PR in one or more lesions in the same patient at a single timepoint during therapy. Inter-organ RDR (RDR occurring between lesions from different organs) and intra-organ RDR (RDR occurring between lesions within the same organs) at each timepoint were recorded. Early RDR was defined as RDR observed in at least one of the first two on-treatment scans, and late RDR was defined as RDR observed in at least one of the final two scans. In patients with three on-treatment scans, early RDR was considered when RDR was observed on at least one of the first two scans and late RDR when it happened on the last one. In patients who only had two or fewer on-treatments scans, the RDR observed in either on-treatment scan was considered as early RDR. Discrepant radiological assessments between both radiologists (RPL and NT) within this study were reviewed anonymously by a multidisciplinary study committee, which comprised both radiologists (RPL and NT) and at least two medical oncologists, with the final consensus radiological assessment recorded.

### *BRCA1/2* mutation status

Patients were classified as germline *BRCA1/2* mutation carriers if a known deleterious mutation in *BRCA1* or *BRCA2* was available through a validated test (Myriad or TruSight panel). If no such mutations were detected with germline molecular testing, the patients were considered *BRCA1/2* wildtype. Patients in whom no germline *BRCA1/2* testing was performed were classified as *“BRCA1/2* status unknown”. Detailed family history of any known cancer was recorded for all patients, as well as a past history of other primary tumors. For the purposes of this study, patients were classified into two categories as either germline *BRCA1/2* mutation carriers, or germline *BRCA1/2* wildtype/unknown (Supplementary Table 1).

### Next generation sequencing (NGS) assays

For the case report detailed in this study, targeted NGS was undertaken on primary and metastatic tumor samples, as well as germline DNA. Somatic DNA from the primary and metastatic tumors was extracted using the formalin-fixed paraffin embedded (FFPE) tissue DNA kit (Qiagen). Germline DNA was extracted from saliva samples using the Oragene Kit. DNA was quantified with the Quant-iT high-sensitivity PicoGreen double-stranded DNA Assay Kit (Invitrogen). Libraries for targeted NGS were constructed from 40ng of DNA using a customized panel (Generead DNAseq Mix-n-Match Panel v2; Qiagen), including all exonic regions of *BRCA1* and *BRCA2*, and analyzed with the Illumina MiSeq Sequencer. FASTQ files were generated using the Illumina MiSeq Reporter v2.5.1.3. Sequence alignment and mutation calling were performed using BWA tools and the GATK variant annotator by the Qiagen GeneRead Targeted Exon Enrichment Panel Data Analysis Web Portal.

### Statistical considerations

The association between baseline characteristics and the presence of RDR was analyzed using 2 statistical tests, the Mantel-Hansel linear-trend test, or the Mann-Whitney U test, as appropriate. Overall survival (OS) was calculated as the time from cycle 1 day 1 until the date of death of the patient from any cause or the date censored at last follow-up. Time to progression (TTP) was calculated as the time from cycle 1 day 1 until the date of disease progression originally recorded in the individual trials. Median survival and TTP rates were estimated using the Kaplan-Meier method, and survival curves generated for each group were compared using the log-rank test. To identify the independent prognostic value of germline *BRCA1/2* mutations, a multivariate analysis (MVA) model was created using a Cox regression model to control the effects of other prognostic variables potentially acting as confounding factors. All p values were two-sided. The SPSS program (version 20.0. SPSS, Chicago, IL) was used for statistical analysis.

## SUPPLEMENTARY MATERIALS FIGURES AND TABLES





## Abbreviations

CR: Complete Response
CT: Computed Tomography
EMA: European Medicines Agency
FDA: Food and Drug Administration
MRI: Magnetic Resonance Imaging
NGS: Next Generation Sequencing
PARP: Poly(ADP-ribose) Polymerase
RDR: Radiological Differential Responses
RECIST: Response Evaluation Criteria in Solid Tumors
SD: Stable Disease
TTP: Time To Progression
OS: Overall survival
PD: Progressive Disease
PR: Partial Response
