# Supplementary material for: High frequency of radiological differential responses with poly(ADP-Ribose) polymerase (PARP) inhibitor therapy
Source: Oncotarget. 2017 Nov 6;8(61):104430–43. doi: 10.18632/oncotarget.22303 (PMC5732817; doi:10.18632/oncotarget.22303)
Supplement: Supplementary file 1 [file oncotarget-08-104430-s001.pdf]

High frequency of radiological differential responses with poly(ADP-Ribose) polymerase (PARP) inhibitor therapy

SUPPLEMENTARY MATERIALS

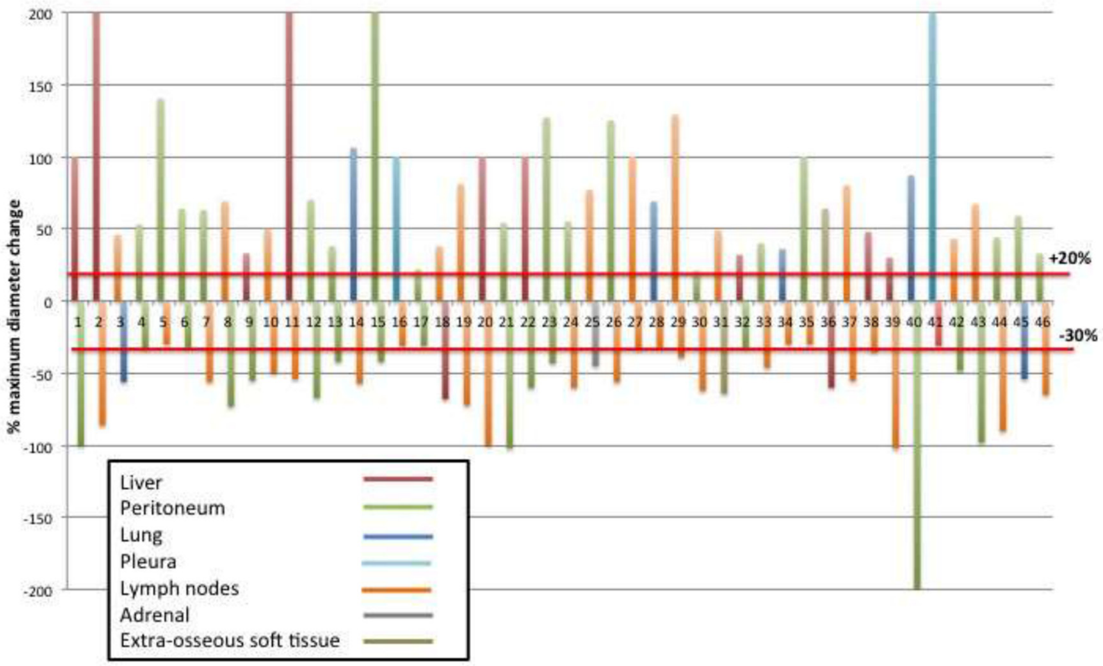

Supplementary Figure 1: Bar chart of the percentage change of diameters of the two lesions with maximum opposite size change per patient with differential response.

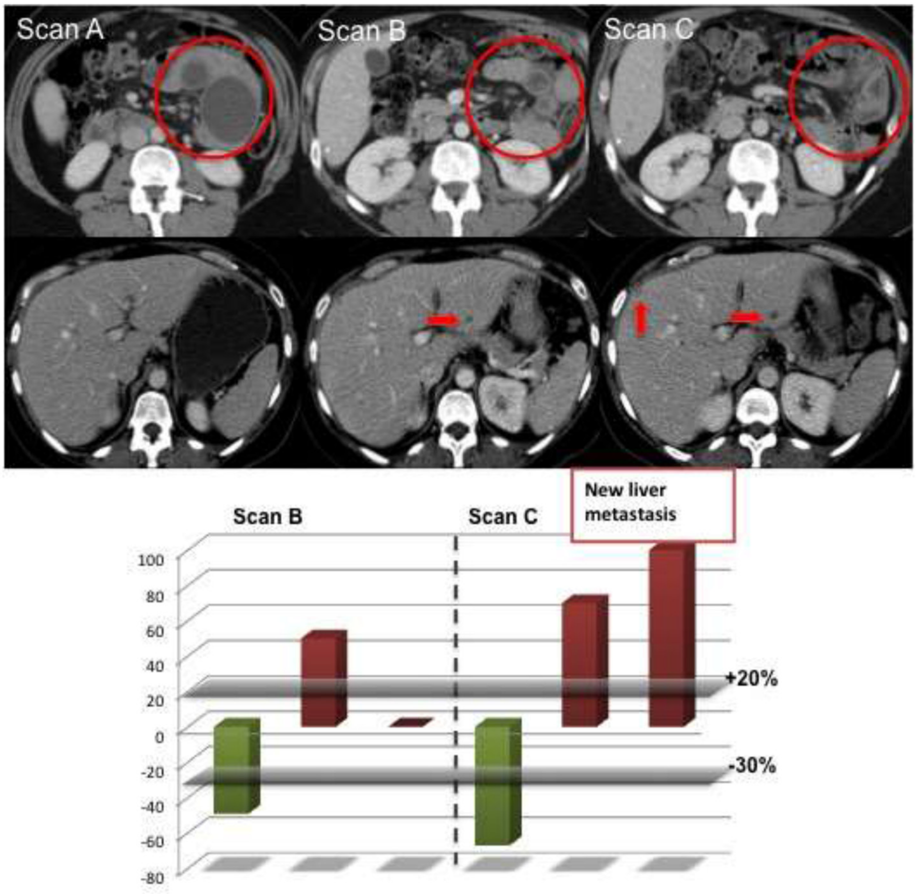

Supplementary Figure 2: Axial enhanced CT images in a 51-year-old female with *BRCA1* mutation ovarian cancer. The baseline **(A)** CT shows a large volume of peritoneal disease with a complex mass involving the small bowel (red circle); no liver metastases were present. The 12 weeks CT **(B)** shows a marked reduction in the size of the complex peritoneal mass (red circle) and new sub centimeter liver lesions, which may potentially represent the emergence of liver metastases (arrow). The 24 weeks CT **(C)** shows an ongoing radiological differential response, with further reduction in the size of the patient's peritoneal disease, with the complex peritoneal mass now barely discernible (red circle), but further increase in size of the liver lesions in keeping with liver metastases (red arrows). The bar chart illustrates the changes in dimensions of the peritoneal and two liver lesions with the maximal alteration in size at each time point.

Supplementary Table 1: Prevalence of RDR in patients with germline *BRCA1/2* mutations compared to patients with wild-type/unknown BRCA1/2 status

gBRCA1/2 mutation carriers	**RDR present**		
	**Yes**	**No**	**Total**
**Yes**	**39 (84.8%)**	**40 (59.7%)**	**79**
**No**	**7 (15.2%)**	**27 (40.3%)**	**34**
**Total**	**46 (100%)**	**67 (100%)**	**113**

Supplementary Table 2: Incidence of RDR in each CT / MRI scan

CT / MRI SCAN	FREQUENCY	INCIDENCE	POPULATION
**First**	**21**	**18.6 %**	**113**
**Second**	**11**	**13.6 %**	**81**
**Penultimate**	**12**	**21.4 %**	**56**
**Final**	**2**	**5.5 %**	**36**

Supplementary Table 3: Baseline characteristics of patients with germline mutation *BRCA1 and BRCA2* mutation cancers. Only the distribution of specific histology subtypes within breast tumors were different between both groups

	BRCA1 **(N=46)**	***BRCA2*** **(N=29)**	**P value**
TUMOR TYPE			
*Ovary	**38 (79.2%)**	**21 (67.7%)**	**p=0.608**
*Breast	**8 (17.0%)**	**8 (25.0%)**	**p=0.57**
PLATINUM SENSITIVITY			
-Refractory	**6 (12.5%)**	**4 (12.9%)**	**p=0.16**
-Resistant	**20 (41.7%)**	**6 (19.4%)**	
-Sensitive	**16 (33.3%)**	**12 (38.7%)**	
HISTOLOGY:			
High grade ovarian	**7 (18%)**	**3 (14%)**	**p=0.56**
ER+ breast	**0 (0.0%)**	**3 (37.5%)**	**p=0.001**
TN breast	**6 (75%)**	**3 (37.5%)**	**p=0.001**
BASELINE DISEASE:			
-Liver	**12 (25.0%)**	**4 (12.9%)**	**p=0.191**
-Peritoneal	**29 (60.4%)**	**20 (64.5%)**	**p=0.718**
-Lymph nodes	**27 (56.3%)**	**20 (51.6%)**	**P=0.818**
NUMBER OF PREVIOUS LINES:	**3.17**	**3.55**	**p=0.579**

Supplementary Table 4A: Location of mutations, tumor type and personal and family history for other cancers among germline *BRCA1/2* mutations carries included in this study.

See Supplementary File 1

Supplementary Table 4B: Tumor type and personal and family history for other cancers among patients who are *BRCA1/2* wild type or with unknown *BRCA1/2* status included in this study

BRCA TESTED	BRCA	TUMOR TYPE	FAMILY HISTORY YN	PLATINUM SENSITIVITY
NO	UK	breast	NO	N/A
NO	UK	breast	NO	N/A
NO	UK	breast	NO	N/A
NO	UK	breast	NO	N/A
NO	UK	breast	NO	N/A
YES	NO	breast	NO	N/A
NO	UK	breast	YES	N/A
NO	UK	cholangiocarcinoma	NO	SENSITIVE
NO	UK	colon	NO	N/A
NO	UK	colorectal	NO	RESISTANT
NO	UK	endometrial	NO	N/A
YES	NO	endometrial	NO	RESISTANT
YES	NO	fallopian tube	NO	SENSITIVE
NO	UK	fibrosarcoma	NO	N/A
NO	UK	leiomyosarcoma	NO	N/A
NO	UK	lung	NO	RESISTANT
NO	UK	lung	NO	N/A
NO	UK	melanoma	NO	N/A
NO	UK	meningioma	NO	N/A
NO	UK	mesothelioma	NO	N/A
NO	UK	ovarian	NO	RESISTANT
NO	UK	ovarian	NO	REFRACTORY
NO	UK	ovarian	NO	RESISTANT
NO	UK	ovarian	NO	REFRACTORY
YES	NO	ovarian	NO	RESISTANT
YES	NO	ovarian	NO	RESISTANT
YES	NO	ovarian	NO	RESISTANT
YES	NO	ovarian	YES	SENSITIVE
YES	NO	ovarian	NO	REFRACTORY
NO	UK	ovarian	NO	SENSITIVE
YES	NO	ovarian	YES	RESISTANT
NO	UK	pancreas	NO	N/A
NO	UK	urethral	NO	N/A
