# Supplementary material for: High frequency of radiological differential responses with poly(ADP-Ribose) polymerase (PARP) inhibitor therapy
Source: Oncotarget. 2017 Nov 6;8(61):104430–43. doi: 10.18632/oncotarget.22303 (PMC5732817; doi:10.18632/oncotarget.22303)
Supplement: Supplementary file 2 [file oncotarget-08-104430-s002.docx]

**Supplementary Table 4A:** Location of mutations, tumor type and personal and family history for other cancers among germline *BRCA1/2* mutations carries included in this study.

| **BRCA** | **MUTATION** | **TUMOR TYPE** | **PLATINUM SENSITIVITY** | **FAMILY HISTORY** | **PREVIOUS CANCER** |
| --- | --- | --- | --- | --- | --- |
| BRCA1 | 5396+1 G>A | OVARIAN | RESISTANT | NO | NO |
| BRCA1 | unavailable | BREAST | NA | YES | YES |
| BRCA1 | c.302-2delA | OVARIAN | SENSITIVE | YES | YES |
| BRCA1 | c.1505_1509delITAAAG | OVARIAN | SENSITIVE | YES | NO |
| BRCA1 | 4184del4 | OVARIAN | SENSITIVE | NO | YES |
| BRCA1 | 6kb insertion | OVARIAN | RESISTANT | YES | YES |
| BRCA1 | 4693delAA | OVARIAN | SENSITIVE | YES | NO |
| BRCA1 | c.3450_3453delCAAG | OVARIAN | SENSITIVE | YES | NO |
| BRCA1 | 5382insC | OVARIAN | RESISTANT | YES | NO |
| BRCA1 | 4184delTCAA | OVARIAN | RESISTANT | NO | YES |
| BRCA1 | 185delAG | OVARIAN | RESISTANT | YES | NO |
| BRCA1 | 5385insc | OVARIAN | RESISTANT | NO | NO |
| BRCA1 | 3875delGTCT | PRIMARY PERITONEAL | RESISTANT | NO | NO |
| BRCA1 | del exon 15-17 | OVARIAN | SENSITIVE | YES | NO |
| BRCA1 | 185AG mutation | OVARIAN | SENSITIVE | NO | NO |
| BRCA1 | 3690ins4 | BREAST | RESISTANT | YES | YES |
| BRCA1 | c.4357+1delG | OVARIAN | REFRACTORY | YES | YES |
| BRCA1 | 5385insC | OVARIAN | RESISTANT | NO | YES |
| BRCA1 | 185delAG | OVARIAN | REFRACTORY | YES | YES |
| BRCA1 | Glycine 1525 argenine FSX5 exon 15 | OVARIAN | SENSITIVE | YES | NO |
| BRCA1 | 6kb insertion | OVARIAN | RESISTANT | YES | YES |
| BRCA1 | unavailable | ENDOMETRIUM | REFRACTORY | YES | NO |
| BRCA1 | 185delAG | OVARIAN | SENSITIVE | NO | NO |
| BRCA1 | 6kb insertion | OVARIAN | SENSITIVE | YES | NO |
| BRCA1 | c.81-2A>G | OVARIAN | RESISTANT | YES | YES |
| BRCA1 | unavailable | OVARIAN | RESISTANT | YES | YES |
| BRCA1 | 1624del5 | OVARIAN | RESISTANT | NO | NO |
| BRCA1 | c.3770_3771delAG | OVARIAN | SENSITIVE | NO | NO |
| BRCA1 | unavailable | BREAST | SENSITIVE | NO | YES |
| BRCA1 | c.1505_1509delITAAAG | OVARIAN | SENSITIVE | YES | YES |
| BRCA1 | del1262 | OVARIAN | RESISTANT | YES | NO |
| BRCA1 | 1624del5 | BREAST | REFRACTORY | YES | NO |
| BRCA1 | c.3005delA | OVARIAN | SENSITIVE | YES | NO |
| BRCA1 | 3452delA | OVARIAN | SENSITIVE | YES | NO |
| BRCA1 | c.3331_3334delCAAG | BREAST | NA | YES | NO |
| BRCA1 | unavailable | BREAST | NA | YES | YES |
| BRCA1 | c.4508C>A | PRIMARY PERITONEAL | REFRACTORY | NO | YES |
| BRCA1 | del exon 1-12 | OVARIAN | RESISTANT | NO | NO |
| BRCA1 | del exon 20 | OVARIAN | RESISTANT | NO | NO |
| BRCA1 | c.3005delA | OVARIAN | RESISTANT | NO | NO |
| BRCA1 | c.3331_3334delCAAG | OVARIAN | RESISTANT | NO | NO |
| BRCA1 | del exon 1-23 | OVARIAN | RESISTANT | YES | NO |
| BRCA1 | unavailable | OVARIAN | RESISTANT | NO | NO |
| BRCA1 | 185delAG | OVARIAN | RESISTANT | NO | NO |
| BRCA1 | 1185C>T (Q356X) | OVARIAN | SENSITIVE | YES | NO |
| BRCA1 | unavailable | BREAST | RESISTANT | NO | NO |
| BRCA2 | 8206T>G | OVARIAN | SENSITIVE | YES | YES |
| BRCA2 | 8206T>G | OVARIAN | SENSITIVE | NO | YES |
| BRCA2 |  | OVARIAN | RESISTANT | NO | NO |
| BRCA2 | c.755_758delACAG | OVARIAN | SENSITIVE | NO | NO |
| BRCA2 | 3715delG | OVARIAN | REFRACTORY | YES | NO |
| BRCA2 | 6174delT | PROSTATE | NA | YES | NO |
| BRCA2 | c.7958A>T | BREAST | NA | YES | NO |
| BRCA2 | 6174delT | OVARIAN | SENSITIVE | NO | YES |
| BRCA2 | c.9253_9254dupA | OVARIAN | NA | YES | NO |
| BRCA2 | 6174delIT | OVARIAN | SENSITIVE | NO | NO |
| BRCA2 | 3715delG | BREAST | NA | YES | NO |
| BRCA2 | c.5130_5133delITGTA | OVARIAN | RESISTANT | YES | NO |
| BRCA2 | 4684del4 | BREAST | NA | YES | YES |
| BRCA2 | 3715delG | OVARIAN | SENSITIVE | YES | NO |
| BRCA2 | 3715delG | BREAST | NA | YES | YES |
| BRCA2 | unavailable | OVARIAN | SENSITIVE | YES | YES |
| BRCA2 | 902delC | BREAST | SENSITIVE | YES | YES |
| BRCA2 | unavailable | OVARIAN | SENSITIVE | NO | YES |
| BRCA2 | c.6275_6276delITT | OVARIAN | RESISTANT | NO | YES |
| BRCA2 | unavailable | OVARIAN | SENSITIVE | YES | YES |
| BRCA2 | c.6275_6276delITT | OVARIAN | RESISTANT | NO | NO |
| BRCA2 | unavailable | BREAST | NA | NO | NO |
| BRCA2 | 8206T>G | OVARIAN | REFRACTORY | NO | NO |
| BRCA2 | c.8575delC | OVARIAN | SENSITIVE | NO | NO |
| BRCA2 | unavailable | OVARIAN | RESISTANT | YES | NO |
| BRCA2 | c.4985C>G | OVARIAN | RESISTANT | YES | NO |
| BRCA2 | c.517-2A>G | BREAST | NA | YES | NO |
| BRCA2 | c.7958A>T | BREAST | NA | UK | UK |
| BRCA2 | c.3778_3779delITT | OVARIAN | SENSITIVE | UK | NO |
| BRCA2 | c.6275_6276delITT | BREAST | NA | YES | NO |
| BRCA2 | c.3778_3779delITT | OVARIAN | SENSITIVE | YES | YES |
| BRCA2 | c.3680_3681delITG | OVARIAN | RESISTANT | YES | NO |
| BRCA2 | c.7988A>T | OVARIAN | RESISTANT | NO | NO |
